# Malnutrition and Gastrointestinal and Respiratory Infections in Children: A Public Health Problem

**DOI:** 10.3390/ijerph8041174

**Published:** 2011-04-18

**Authors:** Leonor Rodríguez, Elsa Cervantes, Rocío Ortiz

**Affiliations:** Departamento de Ciencias de la Salud, Universidad Autónoma Metropolitana-Iztapalapa, Avenida San Rafael Atlixco 186, CP 09340, México, D. F., México; E-Mails: elsi_cervantes@hotmail.com (E.C.); arom@xanum.uam.mx (R.O.)

**Keywords:** gastrointestinal infections, malnutrition, respiratory infections, malnourished children, immune response dysfunction

## Abstract

Infectious disease is the major cause of morbidity and mortality in developing countries, particularly in children. Increasing evidence suggests that protein-calorie malnutrition is the underlying reason for the increased susceptibility to infections observed in these areas. Moreover, certain infectious diseases also cause malnutrition, which can result in a vicious cycle. Malnutrition and bacterial gastrointestinal and respiratory infections represent a serious public health problem. The increased incidence and severity of infections in malnourished children is largely due to the deterioration of immune function; limited production and/or diminished functional capacity of all cellular components of the immune system have been reported in malnutrition. In this review, we analyze the cyclical relationship between malnutrition, immune response dysfunction, increased susceptibility to infectious disease, and metabolic responses that further alter nutritional status. The consequences of malnutrition are diverse and included: increased susceptibility to infection, impaired child development, increased mortality rate and individuals who come to function in suboptimal ways.

## 1. Introduction

Deficiency in macronutrients such as protein, carbohydrates and fat provoke protein-calorie malnutrition (PCM), and when combined with micronutrient deficiencies, they are among the most important nutritional problems with hundreds of millions of pregnant women, elderly and young children particularly affected. Malnutrition is one of the most important underlying causes of child mortality in developing countries, particularly during the first 5 years of life [1]; the major causes for this are poverty, world conflicts, lack of education, natural disasters and poor access to health care.PCM usually manifests early in children between 6 months and 2 years of age and is associated with early weaning, delayed introduction of complementary foods, a low-protein diet and severe or frequent infections [2]. Nearly one-third of children in the developing world are malnourished [3].

Diverse studies have demonstrated that malnutrition increases the risks of infection and death [4,5]. The most frequent causes of death in children under 5 years old are acute diarrhea and acute respiratory infection. Several studies have shown that malnutrition is frequently causally associated with these deaths [6]. However, as malnutrition rarely appears as cause of death on death certificates, its impact is largely underestimated.

Several studies have been conducted to examine associations among malnutrition, deficiencies in cell-mediated immunity, and the incidences of gastrointestinal or respiratory infections in children under 5 years of age. In 2001, the World Health Organization (WHO) established the external Child Health Epidemiology Reference Group (CHERG) to develop estimates of the proportion of deaths in children younger than age 5 years attributable to pneumonia, diarrhea, malaria and measles. Of the estimated 8,795 million deaths in children younger than 5 years worldwide in 2008, infectious diseases caused 68% (5,970 million), with the largest percentages due to pneumonia (18%), diarrhoea (15%), and malaria (8%) [7].A separate study reported different risk estimates, with stronger associations between nutritional status and mortality for gastrointestinal and acute respiratory infections that coincide with malnutrition [8].

The relationship between nutritional status and the immune system has been a topic of study for decades. Several studies have demonstrated that PCM impairs host immune responses, including cell-mediated immunity [9] and secretory IgA production [10,11]. PCM is a major cause of secondary immune deficiency in the world.

In this paper, we focus on describing the association between malnutrition and immune system dysfunction and how this relationship impacts susceptibility to bacterial gastrointestinal and respiratory infections; further, we also discuss the elevated mortality from infectious disease observed in malnourished children. We have reviewed the published literature to identify studies that addressed the relation between malnutrition and mortality from gastrointestinal and respiratory infections. MEDLINE (National Library of Medicine, Bethesda, MD) was searched for original articles using the PUBMED query program. Also we used databases such as EMBASE and Scopus. These databases were searched from 1950 up to 2010 for literature published either in English, Spanish or in a foreign-language publication with an English abstract.

Combinations of the following groups of keywords were used: malnourished children, malnutrition, protein energymalnutrition, gastrointestinal infections, respiratory infections, pneumonia, and immune response. Then a separate search was conducted to identify publications related to the immune response *versus* the main etiologic agents of gastrointestinal and respiratory infection in children with malnutrition.

In each section of this review, gastrointestinal infections and respiratory infections are presented separately. Studies that examined the relation between malnutrition and other types of pathogenic agents (for example, infections associated with HIV or other viruses), were not included in this review. Also, studies that were conduced in adult malnourished were excluded.

## 2. Malnutrition

Nutritional status affects every aspect of a child’s health, including normal growth and development, physical activity, and response to serious illness. Malnutrition may originate from the deficiency or absence of any nutrient. The establishment and severity of malnutrition depends on the cause, intensity and duration of the nutritional deficiency. It can be caused, primarily, by an inadequate diet or, secondarily, by deficiency in gastrointestinal absorption and/or increase in demand, or even, by an excessive excretion of nutrients [12]. Protein-calorie malnutrition (PCM), also known as protein-energy malnutrition, is defined by the WHO as being a pathological condition that results from a lower ingestion of protein and calories, which occurs more frequently in children under five years of age.

**Figure 1.**
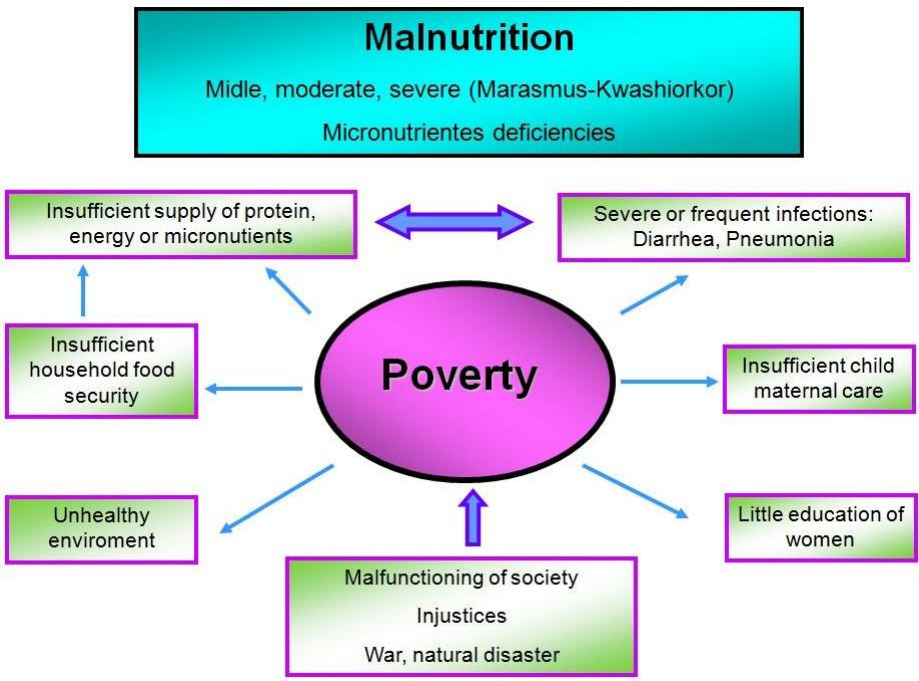
Direct and indirect causes of malnutrition, showing that poverty is the main underlying cause of malnutrition and its determinants. Adapted from Müller and Krawinkel [14].

Figure 1 shows the direct and indirect causes of malnutrition. It is important to reflect on the thoughts of Joaquin Cravioto, a prominent Mexican nutritionist: “The basic origin of malnutrition is to be found in the malfunctioning of society as a whole and the accompanying injustices” [13].

In 2009, the WHO estimated that 27% of children in developing countries under the age of 5 years are malnourished. Approximately 178 million children (32% of children in the developing world) suffer from chronic malnutrition. Although the prevalence of childhood malnutrition is decreasing in Asia, countries in South Asia still have both the highest rates of malnutrition and the largest numbers of malnourished children. Indeed, the prevalence of malnutrition in India, Bangladesh, Afghanistan, and Pakistan (38–51%) is much higher than in sub-Saharan Africa (26%) [3]. In Mexico, the most recent national nutrition survey estimated that 1.8 million children under 5 years of age are malnourished [15].

Malnutrition is diagnosed by anthropometric measurements and physical examination. Correlation of malnutrition and growth retardation allows assessment of the individual nutritional state, which is usually measured as body mass index (BMI). BMIs are given as weight-for-height [16]. PCM is defined by measurements that fall below 2 standard deviations under the normal weight-for-age (underweight), height-for-age (stunting) and weight-for-height (wasting) [17]. Wasting indicates recent weight loss, whereas stunting usually results from being chronically underweight. Of all children under 5 years of age in developing countries, about 31% are underweight, 38% have stunted growth and 9% show wasting [14].

Underweight, stunting, and wasting forms PCM each represent different histories of nutritional deficits. Occurring primarily in the first 2–3 years of life, linear growth retardation (stunting) is frequently associated with repeated exposure to adverse economic conditions, poor sanitation, and the interactive effects of poor energy and nutrient intake and infection. Low weight-for-age indicates a history of poor health or nutritional deficiencies, including recurrent illness and/or starvation. In contrast, low weight-for-height is an indicator of wasting or thinness and is generally associated with recent illness, weight loss or a failure to gain weight [18].

In addition, malnutrition is frequently classified on the basis of deficits of weight-for-age (w/a) or height-for-age [19,20]. In this system, children are classified into three groups according to malnutrition severity based on their weight compared to the weight average for their age. First degree or mild cases of malnutrition include children whose weights are 76–90% of the average weight. Children with second degree or moderate cases have weights between 61–75% of the average, and children with third degree or severe malnutrition weigh 60% or less than their peers [19]. With time, the so-called “Gómez classification” has been used widely both to classify individual children for clinical referral and to assess malnutrition in communities [21]. The stratification of malnutrition as mild, moderate or severe has helped to systematize clinical observations and has allowed for the comparison of findings between different researchers [13]. Moreover, the risk of death is directly correlated with the degree of malnutrition [22]. In developing countries, about 3.5% of children under the age of 5 years suffer from severe malnutrition. Although mild and moderate types of childhood malnutrition are even more prevalent, their significance in childhood morbidity and mortality is less well recognized [3].

Severe PCM appears in three principal clinical forms: (1) marasmus, characterized by chronic wasting condition and a gross underweight status that is habitually associated with early weaning; (2) kwashiorkor, characterized by moderate growth retardation, changes to hair and skin color, edema, moon facies, and hepatosplenomegaly; and (3) marasmic kwashiorkor, characterized by severe wasting and the presence of edema. Marasmus appears by caloric and protein insufficiency, whereas kwashiorkor develops from protein deficiency [23].

Epidemiological and experimental observations have proven that malnourished children are more susceptible to infectious disease; therefore, PCM is considered a strong risk factor for higher morbidity and mortality rates in infectious disease [24]. Several studies on the effects of malnutrition at the immunological level have been conducted in humans and in experimental animal models. Multiple immune system abnormalities, including lymphoid organ atrophy, profound T-cell deficiency, altered ratios of T-cell subsets, and decreased natural killer (NK) cell activity and cytokine production have been described in PCM individuals. In addition, these studies indicate that malnutrition decreases T-cell function, cytokine production and the ability of lymphocytes to respond appropriately to cytokines. In severely malnourished children, both acquired immunity as well as innate host defense mechanisms are affected [25–27].

**Figure 2.**
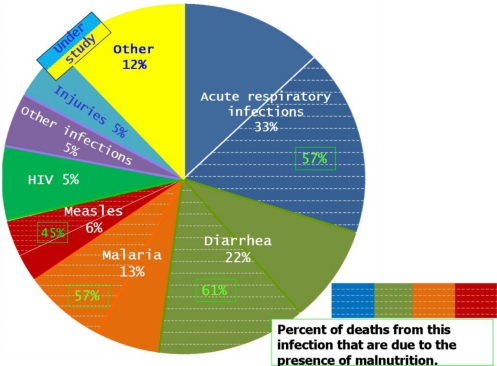
Distribution of 10.5 million deaths among children younger than 5 years of age in all developing countries. Adapted from Benguigui and Stein [28].

In children under 5 years of age, malnutrition is responsible, directly or indirectly, for 54% of the 10.8 million deaths per year and contributes to every second death (53%) associated with infectious disease among this age group in developing countries (Figure 2) [28]. Additionally, mild and moderate forms of malnutrition primarily account for the burden of malnutrition worldwide. For the surviving children, malnutrition has lifelong implications because it severely reduces a child’s ability to learn and grow to their full potential. Thus, malnutrition leads to less productive adults and weaker national economic performance [28].

The malnutrition-infection complex can be viewed under two aspects, malnutrition compromising host defense, or infection either aggravating a previously existing deficient nutritional status or triggering malnutrition through disease pathogenesis. Malnutrition can facilitate pathogen invasion and propagation; further, it can increase the probability of a secondary infection occurring, thus modifying both disease pathogenesis and prognosis [29].

Certain infectious diseases also cause malnutrition. It appears that there is a vicious cycle involved, where malnutrition increases disease susceptibility and disease causes a reduction in food intake. The relationships among malnutrition, immune suppression and infection are complicated by the severe effects that a number of infections exert on nutrition. Examples of how infections can contribute to malnutrition include: (1) gastrointestinal infection that lead to diarrhea, (2) chronic infections that cause cachexia and anemia; and (3) intestinal parasites that cause anemia and nutrient deprivation [16].

Acute diarrhea and pneumonia occur most frequently during the first 2–3 years of life when immunocompetence is impaired and when children are first being exposed to pathogens. Infection can suppress appetite and directly affect nutrient metabolism, leading to poor nutrient utilization [18].

## 3. Immune System

The immune system is capable of mounting effective immune responses to an almost infinite variety of foreign pathogens or tumor cells, while avoiding harmful immune responses to self. This system consists of a sophisticated array of cells that have developed mechanisms to both recognize and eradicate a wide variety of pathogenic microorganisms [30].

Both innate immunity and adaptive immune responses depend upon the activities of white blood cells, or leukocytes. Innate immune defenses are those components of the immune system, such as macrophages, monocytes, and neutrophils that function without requiring previous exposure to a particular antigen. Adaptive or acquired immune responses develop in response to specific antigens and pathogens and exhibit memory qualities, rapidly responding if the antigen or pathogen is encountered again in the host’s lifetime. The combination of these two systems defends the host against infection. Innate immunity provides a first line of defense against pathogens and can be activated rapidly following infection; this response is non-specific and involves epithelial barriers, circulating phagocytes (mainly neutrophils and macrophages), and other cytotoxic cells, such as NK cells; further, complement proteins and positive acute-phase proteins (APP) also play a role [31,32].

In the first part of an immune response, the defenses of the body include the epithelial cells that line the internal and external surfaces of the body and the phagocytes that can engulf and digest invading microorganisms. In addition to killing microorganisms, several phagocytes also induce the next phase of the early response, and, if the infection is not cleared, they also activate adaptive immune responses [33]. APP are regulated by proinflammatory cytokines produced primarily by macrophages and neutrophils, such as IL-1, TNF-α, IL-6, and IL-12, as well as anti-inflammatory cytokines, such as IL-10, which down-regulate inflammation once pathogens have been eliminated [34]. Central to the development of an organized host cellular response to infection is the recruitment of immune effector cells, such as neutrophils, monocytes, and lymphocytes to the site(s) of infection. In recent years, a large number of signaling molecules, which have come to be known as chemokines, have been identified as key molecules in recruiting immune cells [35].

## 4. Relationship between Malnutrition and Infection

**Figure 3.**
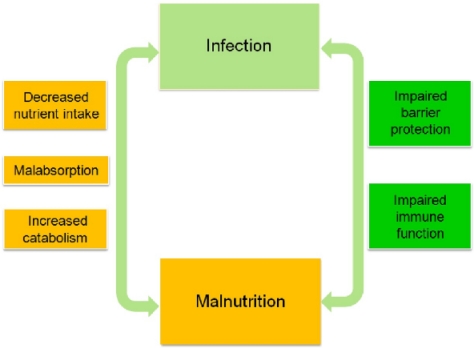
Relationship between nutrition and infection. Adapted from Brown [37].

A great number of field studies have demonstrated that the relationship between infection and malnutrition is bidirectional (Figure 3) [36,37].The site of interaction as well as the type of pathogen can largely determine which type of immune response will proceed, and whether it will be an optimal response. Initiation of both innate and adaptive immune responses involves the activation and proliferation of immune cells and the synthesis of an array of molecules; the associated DNA replication, RNA expression, protein synthesis and protein secretion consumes additional anabolic energy. Consequently, the nutritional status of the host critically determines the outcome of infection [16].

**Figure 4.**
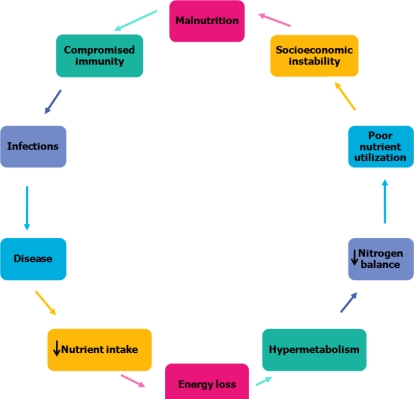
Protein Energy Malnutrition Increases Prevalence of Infection, Leading to Energy loss for the Individual. Adapted from Schaible *et al.* [16].

There are multiple mechanisms of action in the relationship between malnutrition and susceptibility to bacterial infections diseases. For instance, PCM impairs normal immune system development [26]. Stimulation of an immune response by infection increases the demand for metabolically derived anabolic energy, leading to a synergistic vicious cycle of adverse nutritional status and increased susceptibility to infection (Figure 4). Infection itself can cause a loss of critical body stores of protein, energy, minerals and vitamins. During an immune response, energy expenditure increases at the same time that the infected host experiences a decrease in nutrient intake [38]. The metabolic response to infection includes hypermetabolism, a negative nitrogen balance, increased gluconeogenesis and increased fat oxidation, which is modulated by hormones, cytokines and other pro-inflammatory mediators [39]. During an infection, a negative nitrogen balance occurs after fever induction and then it increases and persists for days to weeks after the febrile phase. Additionally, negative nitrogen balance appears to correlate with net loss in body weight; both conditions are the result of reduced food intake and infection induced-increased nitrogen excretion [40,41].

Malnourished children suffer in greater proportion from bacterial gastrointestinal and respiratory infections [42]. The first line of defense against these types of infection is the innate immune response, particularly epithelial barriers and the mucosal immune response [34]. PCM significantly compromises mucosal epithelial barriers in the gastrointestinal, respiratory and urogenital tracts. For example, vitamin A deficiencies induce the loss of mucus-producing cells. This loss of the protective mucus blanket increases susceptibility to infection by pathogens that would ordinarily be trapped in the mucus and swept away by the cleansing flow of mucus out of the body. Barrier defects of mucous membranes are critical in the pathogenesis of respiratory and gastrointestinal tract infections [38].

In particular, mucosal barrier immunity is impaired in the malnourished host in the gastrointestinal tract due to the altered architecture and composition of the intestinal mucosal tissues which includes flattened hypotrophic microvilli, reduced lymphocyte counts in Peyer’s patches or reduced IgA secretion [43]. Secretory IgA is an important component of the mucosal immune response that protects the upper respiratory and gastrointestinal tracts against infection with pathogenic organisms.

Previously, it has been reported that total IgA concentration is reduced in the intestinal mucosa of protein-malnourished mice [44,45]. The authors suggest that protein malnutrition may decrease IgA content by suppressing the proliferation and/or maturation of IgA-producing B-cells. Additionally, studies have shown that protein malnutrition suppresses the expression of the epithelial IgA-transporting protein, which decreases the total IgA concentration in the intestinal lumen [46]. Thus, PCM appears to impair IgA-dependent mucosal immune defenses, including the production of IgA by plasma cells and its secretion into the lumen of the intestine [45].

In protein-malnourished mice, significantly decreased levels of IL-4 were reported in the small intestinal mucosa. Interestingly, these findings correlated with reduced secretory IgA production [45]. Malnourished mice, which are more susceptible to infection, exhibit altered innate immune responses and decreased nitric oxide production from resident peritoneal macrophages compared to control mice [47].

The level and features of the APP response are dependent on host nutritional state and infection severity [48]. Severe malnutrition affects the APP response by reducing the availability of precursors for APP synthesis or by reducing the synthesis of modulating proinflammatory cytokines such as IL-1 and IL-6. Proinflammatory cytokines responses during the acute phase of infection are affected by malnutrition. Specifically, serum IL-1 concentrations are markedly lower in infected, malnourished children compared to infected, well-nourished children [49]. It has been reported that severely malnourished children mount only a partial APP response to the infection, particularly; children with edematous malnutrition had higher plasma concentrations of C reactive protein, α-1-antitrypsin and haptoglobin [50].

Complement, another element of the innate immune response, is also altered during malnutrition. Specifically, serum levels of C3 tend to be decreased in severely malnourished children compared to normal children [51]. As the initial events in phagocytosis and microbial killing are largely complement dependent, this deficiency resulted in a significant impairment in leukocyte microbicidal capacity early in infection, which was particularly evident for gram-negative organisms [26].

Additionally, serum levels of leukotrienes, which enhance leukocyte accumulation and phagocyte capacity, have been reported to be markedly diminished in children with PCM. For example, decreased leuokotriene levels were associated with reduced microbial ingestion and killing by phagocytic cells [52]. Moreover, it has been reported that experimental malnutrition impairs leukocyte exudation into local inflammatory sites by reducing production of the chemokine macrophage inflammatory protein [53]. In addition to decreased chemokine production, there is a decrease in the functionality of the chemokine that is produced; combined, these factors can result in an inadequate inflammatory response.

The changes in mucosal immune function presumably account for the increased mortality seen in malnourished children. Therefore, PCM may increase susceptibility to gastrointestinal and respiratory infections, possibly as a result of impaired mucosal immune response and/or systemic alterations of immune response.

## 5. Gastrointestinal Infections Associated with Malnutrition

PCM and gastrointestinal bacterial infections frequently coexist in humans living in developing countries. It is estimated that more than 10 million children under 5 years of age die each year worldwide [54]. More than two million children die each year in developing countries from diarrheal diseases. Infection adversely affects nutritional status through reductions in dietary intake and intestinal absorption, increased catabolism and sequestration of nutrients that are required for tissue synthesis and growth [55].

Of 3 million premature deaths due to diarrheal diseases, approximately 58% are associated with malnutrition [56]. The close relationship between diarrheal disease and malnutrition has not escaped the attention of the scientific community. Global estimates for mortality from diarrheal diseases have declined from approximately 4.6 million annual deaths during the mid-1980s to the current estimate of 1.6–2.1 million. However, although rates of mortality from diarrhea have decreased, morbidity rates remain as high as ever [57].

In a recent descriptive and prospective study, 335 children under 6 years of age that were admitted to a hospital in Colombia for severe acute malnutrition (83%) or moderate acute malnutrition associated with illness (17%). The most common complication upon admission was diarrhea (68.4%) and the most common complication during hospitalization was sepsis (9%). Children with moderate acute malnutrition had similar complications and mortality when compared to children with severe acute malnutrition [58].

The epithelium of the gastrointestinal tract is formed by a single layer of cells. This biological structure separates the intestinal lumen from the internal body, functioning as the intestinal barrier. It regulates important functions such as intestinal digestion, secretion, and absorption of nutrients [59].

Gastrointestinal infections impair weight and height gains and physical and cognitive development. Mechanistically, these outcomes have been attributed to damage to the mucosal barrier and villus atrophy, which reduces nutrient absorption. In PCM, decreased intestinal villus heights have been observed, mostly likely caused by a significant reduction in enterocyte numbers and proliferation. Overall, these changes resulted in decreases in total surface area and mucosal mass [60,61]. Several reports suggest that these lesions continue throughout childhood and into adulthood [62]. Furthermore, moderate-to-severe malnutrition alone can alter villus and crypt architecture [63]. Malnutrition can also increase lamina propria macrophage and lymphocyte populations and proinflammatory cytokine production in the intestinal mucosa, which may further alter intestinal barrier function [64].

The rate of protein turnover in the gut mucosa is very high and is therefore sensitive to changes in host nutritional status [65]. Welsh *et al.* [66] reported a significant increase in intestinal permeability in malnourished children that was associated with activation of lamina propria mononuclear cells and enterocytes, leading the authors to conclude that intestinal barrier function is significantly compromised in malnourished patients. Further, abnormal intestinal permeability in kwashiorkor malnourished children was associated with diarrhea, sepsis, and death. Diarrhea and death were associated with both decreased absorption due to diminished absorptive surface area, and increased intestinal permeability caused by impaired barrier function [67]. Interestingly, an increased inflammatory state in the lamina propria might also impair intestinal barrier function and ultimately lead to increased intestinal permeability and weight and height growth deficits in children [68].

The gastrointestinal associated lymphoid tissues (GALT) comprise a secondary lymphoid tissue where effector immune responses directed gastrointestinal pathogens occur. Peyer’s patches, an example of GALT, are aggregates of lymphoid follicles located along the small intestinal mucosa that protects the body; they respond to antigens that have passed through mucosal surface barriers [69].

Structurally, Peyer’s patches contain proliferating B-lymphocytes, dendritic cells, macrophages and T-cells. Antigens in the lumen of the gut are transported to the Peyer’s patches and initiate the immunologic response. This response is principally mediated by IgA production from activated B lymphocytes. This secretory IgA is released into the intestinal lumen. The main function of secretory IgA is to neutralize foreign pathogens by preventing binding to and penetration of epithelial cells. Moreover, the secreted cytokines for the epithelial barrier, part of mucosal immunity, regulate local immune responses [69]. Gut mucosal immunity is very susceptible to PCM, this is associated with dysregulated cytokine production [70].

In children, malnutrition increases both the frequency (37%) and duration (73%) of diarrheal illnesses, resulting in a doubling of the diarrhea burden (days of diarrhea) [71]. In contrast, other work has showed that nutritional status may not play an important role in increasing the susceptibility of children to diarrhea [72]. It has been proposed that children in poor communities are malnourished because they do not get enough food, not because they suffer from diarrhea [73]. However, when the interrelationship between diarrhea and malnutrition was investigated in a population with moderate malnutrition, both low weight-for-age and diarrhea itself are associated with increased diarrhea risk [74].

Gastrointestinal infections, such as diarrhea and gut helminth infections, directly affect the integrity, morphology, and function of the absorptive mucosa of the intestine possibly resulting in malabsorption [75]. It is has been propose that an important proportion of childhood malnutrition is due to impaired intestinal absorptive function resulting from multiple and repeated gastrointestinal infections [76]. Malnutrition can cause blunting of the villus architecture and a reduction in the brush border, which ultimately results in nutrient malabsorption and a further decline in nutritional status if not treated appropriately [3]. A proposed mechanism whereby diarrhea causes malnutrition includes metabolic changes derived from infection and/or intestinal malabsorption [77].

A direct correlation between malnutrition severity and the magnitude of decrease in lactase, and maltase activities has been reported. [61]. Moreover, it has been shown that changes in the microvillous membrane of the small intestine are related to alterations in carbohydrate and lipid absorption. This phenomenon results from the malnutrition-induced diminished activity of disaccharidase and dipeptide hydrolase, enzymes that located in the intestinal microvillous membrane. Therefore, the malabsorption of dipeptides and disaccharides might contribute to diarrhea and growth failure in malnourished children [78].

Lactose is the major source of dietary carbohydrate during infancy; therefore, the effect of malnutrition on mucosal lactase specific activity is of particular importance during this time period [78]. A significant reduction in lactase activity in patients with malnutrition has been reported [79].

In relation to metabolic changes, acute infections cause anorexia and decrease nutrient intake. Studies show that children with diarrhea consumed 18% less calories per day compared to healthy children [80]; this impact becomes more distinct the more severe the infection. Furthermore, recent reviews demonstrate that metabolic changes in PCM include amino acid and protein deficiencies, carbohydrate and energy deficiencies, hypolipidemias, hypolipoproteinemias, hormonal imbalance and deficiencies of anti-oxidant vitamins and enzymes [81].

*Helicobacter pylori* is a causative agent of disease states of varying severity including chronic gastritis, or gastric adenocarcinoma [82]. *H. pylori* infection is strongly associated with other gastrointestinal infections and chronic malnutrition. *H. pylori* infection occurs primarily in early childhood, and in developing countries it has a severe impact on general health [83]. In children, *H. pylori* infection can be the initiator of a vicious cycle of events than leads to malnutrition and growth retardation in children that impacts both morbidity and mortality [83,84].

Several studies show an association between acute *H. pylori* infection and transient or extended periods of hypochlorhydria (*i.e.*, reduction in gastric acid secretion) in children. Furthermore, other data showed that *H. pylori*-infected children have impaired gastric acid secretion [85,86], which can provoke diarrhea [83].

Gastrointestinal pathogenic bacteria can either be ingested or ascend from the distal bowel; however, their survival is usually limited by gastric acidity. Therefore, the hypochlorhydria can result in bacterial overgrowth in the stomach; further, the expanded bacterial populations may also contribute increasing the intragastric pH [87]. Also, hypochlorhydria increases susceptibility to enteric infections such as salmonellosis, cholera, giardiasis, Shigellosis, and others due to the loss of the gastric acid barrier [83,88].

A combination of PCM and coinfection with enteropathogens that provoke diarrhea acquired as a consequence of *H. pylori*-induced hypochlorhydria is likely to have a profound impact on pediatric populations where the prevalence of *H. pylori* infection is high [83]. Indeed, the incidence of *H. pylori* infection in malnourished children is greater than in well-nourished children [89], due to high IL-1 production that is associated with hypochlorhydria that favors chronic *H. pylori* infection [82].

Acid concentrations and gastric juice secretion rates are diminished in severely malnourished children [75]. Further, these children exhibited elevated levels of bacterial colonization associated with the reduced gastric acid barrier. These data suggest that the gastric acid barrier may be a protective factor in children [90]. Therefore, hypochlorhydria may allow subsequent bacterial infection of the upper gastrointestinal tract in severely malnourished children [90,91]. Infection of the intestinal tract with several well known bacterial pathogens can profoundly disrupt intestinal function with or without causing overt dehydrating diarrhea. Diarrhea is a syndrome that is frequently not differentiated clinically by specific etiologic agent. In addition, diarrhea is a frequent complication of malnutrition [78,92–94]. Additionally, malnutrition is considered a host factor that influences susceptibility to amebiasis [95].

Mondal *et al.* [96] investigated the association of gastrointestinal infection-induced episodes of diarrhea with the nutritional status of children. They concluded that amebiasis, caused by the invasion of the intestinal wall by the protozoan parasite *Entamoeba histolytica,* is strongly associated with a high incidence of diarrhea in malnourished children. *E. histolytica* infection results from ingestion of the parasite through fecal-contaminated food or water. In developing countries, infection with *E. histolytica* has been observed in 2–10% of diarrheal episodes in children. *E. histolytica*-induced amebiasis is estimated to result in 50 million infections and 100,000 deaths worldwide each year [97]. Secretory IgA antibodies are associated with protective immune responses against *E. histolytica* diarrhea and colonization [98,99]. Therefore, the increased incidence of *E. histolytica* in malnourished children may result from the significantly decreased number of IgA-secreting cells present in the small intestine lamina propria of malnourished children [100].

Other responses against *E. histolytica* include innate immune responses. Macrophages are central to innate and acquired immune responses; they are activated by a variety of stimuli. IFN-γ induces the differentiation and activation of monocyte-macrophages and enhances their microbicidal activity [101,102]. Particularly, IFN-γ activates macrophages to kill *E. histolytica in vitro*; consistent with this, mice that are susceptible to amebiasis showed deficient IFN-γ production [95]. The effects of malnutrition on macrophage function have been reported in several studies [103,104]. In relation to has been demonstrated that malnutrition results in impaired macrophage phagocytosis, impaired production of superoxide anion and reduced cytokine production [105]. Additionally, data from our previous study showed a significant decrease in IFN-γ production by CD4+ and CD8+ T-cells from malnourished children [27].

Additionally, well-nourished children colonized with *E. histolytica* showed more IFN-γ production than healthy well-nourished children without infection. Therefore, the authors concluded that IFN-γ was associated with protection from *E. histolytica* infection [95]. Consistent with these findings, PBMC from malnourished children stimulated with soluble amebic antigen exhibited significantly lower production of IFN-γ compared to well-nourished children [106]. Therefore, they concluded that the susceptibility of malnourished children to amebiasis might be explained, at least in part, by a deficiency in the ability of their cells to produce IFN-γ in response to amebic antigen. Decreased gastric acidity accompanied by a specific decrease in IFN-γ production in malnourished children, suggests that malnutrition may predispose children to amebiasis by suppressing normally protective cell-mediated immune responses.

A broad group of microorganisms cause diarrhea in children making identification of the etiologic agent difficult. Bacterial enteric pathogens that cause most cases of severe acute diarrhea include *Vibrio cholerae*, *Shigella* spp., *Salmonella* spp., enteropathogenic *Escherichia coli* (EPEC), enteroaggregative *E. coli* (EAEC), enterotoxigenic *E. coli* (ETEC) and *Cryptosporidium* spp. [96,107–110]. Furthermore, intestinal helminth infections may also impair intestinal function, absorption and growth [111,112].

CHERG has also estimated morbidity from specific enteric pathogens based on broad reviews of studies that have documented the etiologic agents of diarrhea. The most frequent bacterial etiologies of diarrhea at the community level were ETEC (14%) and EPEC (9%). Although *Campylobacter* spp. (12.6%) and EPEC (9%) were most frequent in outpatient studies, EPEC (16%) and ETEC (9%) were the most frequent species in inpatient studies. The CHERG findings also suggest that much more morbidity than mortality is caused by certain enteric pathogens such as *G. lamblia*, *Cryptosporidium* spp., *E. histolytica*, and *Campylobacter* spp.; in contrast, enteric pathogens such as rotavirus, *Salmonella* spp. and *V. cholerae* seem to be important causes of mortality [113].

*Cryptosporidium* spp. and EAEC modify and provoke mucosa inflammation, also cause disease mainly by inducing host production of cytokines. Also, EPEC induces important changes in epithelial cell function [113]. Intestinal infections with *Salmonella* spp. and *Shigella* spp. also activate the production of cytokines and chemokines that cause inflammation that affects intestinal epithelial cell function [114]. Specifically, *Shigella* spp. invades intestinal epithelial cells, which results in barrier disruption and inflammation [115].

In relation to cytokines, several studies indicate that malnutrition decreases T-cell function, cytokine production, and the ability of lymphocytes to respond appropriately to cytokines [27,116,117]. Malnourished children have been shown to have altered capacities to produce several cytokines (*i.e.*, IL-2, IL-4, IL-6, IL-10, *etc.*). González *et al.* [118] observed that lymphocytes obtained from malnourished children were unable to secrete normal quantities of cytokines or to achieve adequate immunologic function and proposed that the altered physiology of lymphocytes may be the predominate cause of the immune impairment observed in malnourished children.

## 6. Respiratory Infections Associated with Malnutrition

A strong and consistent association has been demonstrated between malnutrition and mortality from respiratory infections; further, malnutrition is considered to be a more important risk factor for pneumonia than for diarrhea [119,120]. Acute respiratory infections (ARIs) are the leading cause of high mortality and morbidity among children under 5 years of age [121]; they are also the most frequent cause of health services used around the world. ARIs represent between 30–50% of pediatric medical consultations and between 20–40% of hospitalizations in children. The risk factors for acquiring respiratory infections are poverty, restricted family income, low parental education level, lack of breastfeeding and, most importantly, malnutrition [122].

As above mentioned, the establishment of malnutrition depends on the cause and duration of the any nutritional deficiency. It can be caused, secondarily, by increase in demand of nutrients [12].

The infection may be either aggravating a previously existing deficient nutritional status or triggering malnutrition through disease pathogenesis [29]. It has been demonstrated that certain infectious diseases cause malnutrition. These diseases cause a reduction in food intake. One example of how respiratory infections can contribute to malnutrition is that chronic infections may be cause cachexia [15]. The respiratory infections, as pneumonia, occur most frequently during the first 24–36 months of life when immunocompetence is impaired and when children are first being exposed to pathogens. The stimulation of an immune response by respiratory infection increases the demand for metabolically derived anabolic energy, this lead to adverse nutritional status. Moreover, a respiratory infection itself can cause a loss of critical body stores of protein and energy. During an immune response, energy expenditure increases at the same time that the infected host experiences a decrease in nutrient intake [37]. Additionally, negative nitrogen balance appears to correlate with net loss in body weight; this result in reduced food intake and infection induced-increased nitrogen excretion [39,40]. During an infection, a negative nitrogen balance occurs after fever induction and then it increases and persists for days to weeks after the febrile phase. Therefore, the malnutrition may be a consequence of repeated respiratory infections, common in young children [123].

The incidence of *Streptococcus pneumoniae* in children younger than 5 years of age in developing countries varies greatly [122]. In developing countries, more than 8,795 million children die each year. In 2008, more than 5,970 million children died due to infectious diseases; approximately 18% (1,575 million) of these deaths were caused by pneumonia [7]. In contrast, other data show that there are more than 9 million deaths among children under the age of five globally each year, of which, about three million deaths are due to pneumonia [55,124]. Regardless of the total numbers, the majority of ARI-related deaths occur in developing countries. Although these numbers represent the most rigorous estimate of child deaths caused by *S. pneumoniae*, they are probably an underestimate [125].

In America, approximately 100,000 deaths per year caused by ARI in children under 1 year of age have been reported since the 1980s. Five countries contributed to 85% of these deaths: Brazil (40%), Mexico (19%), Peru (14%), Bolivia (7%) and Haiti (5%). The Pan American Health Organization (PAHO) estimates that the percentage of deaths attributed to ARI varies from 2% to 16%. Meanwhile, in countries such as Canada and the United States, the percentage of deaths attributed to ARI in this age group is 2% [122].

**Figure 5.**
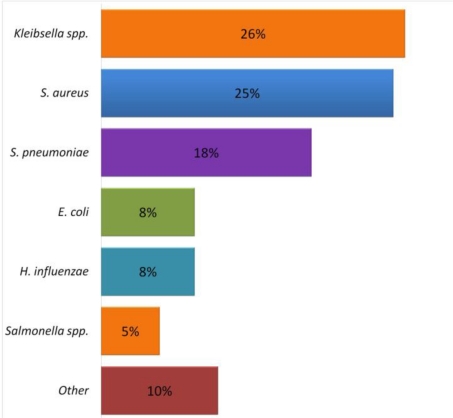
Agents pathogens in children with pneumonia and severe malnutrition in developing countries. Adopted from Chisti *et al.* [130].

Childhood clinical pneumonia is caused by a combination of risk factors related to the host, the environment and infectious agent [126]. In developing countries, identifying the etiology is difficult, and WHO recommends diagnosing pneumonia based on clinical parameters. However, based on available evidence, several studies have identified *Streptococcus pneumoniae* and *Haemophilus influenzae* as the most important pathogens associated with childhood pneumonia [127,128]. Further, *Staphylococcus aureus* and *Klebsiella pneumoniae* have also been linked to cases of severe pneumonia [129]. In microbiologic studies, *Streptococcus pneumoniae* has been identified in 30–50% of pneumonia cases and *H. influenzae* type b in 10–30% of cases. *S. aureus* and *K. pneumoniae* were the next most prevalent etiologic agents of pneumonia [126]. However, with the increased use of pneumococcal and *H. influenzae* type b vaccines in developing countries, it is likely that these pathogens will become relatively less important as causative agents of pneumonia [130]. Bacterial pathogens in children with pneumonia in developing countries obtained from several studies are shown in Figure 5.

*Streptococcus pneumoniae* is a leading cause of bacterial pneumonia, meningitis, and sepsis in children worldwide. Pneumococcal disease is preceded by asymptomatic nasopharyngeal colonization, which is especially high in children. The natural route of infection with *S. pneumoniae* starts with colonization, which may progress to invasive disease if immunological barriers are crossed [131].

*Haemophilus influenzae* type b (Hib) is mostly an opportunistic pathogen that causes invasive infections, such as pneumonia in children under 5 years of age. The incidence of Hib pneumonia and Hib invasive disease in children younger than the age of 5 years in developing countries is 7 and 21–60 per 100,000 per year, respectively [132,133]. Rudan *et al.* [126] reported that in developing countries with a high burden of pneumonia, 15–30% of radiological pneumonia cases, and most likely the same proportion of pneumonia deaths, is due to Hib.

Up to two-thirds of malnourished children that are hospitalized are diagnosed with pneumonia [134]; generally, the etiologic agent is *S. pneumoniae*. Despite the availability of antibiotics, mortality and morbidity rates remain high, especially in high-risk groups like malnourished children [135]. Pneumonia is common in malnourished children and is frequently associated with fatal outcome [136], especially in malnourished children younger than 24 months of age [137]. Although ARIs are caused by a wide variety of bacterial agents, studies consistently reported a two- to threefold greater risk of mortality associated with malnutrition [138]. Therefore, pneumonia and malnutrition are two of the biggest killers in childhood disease [130].

A recent study that described clinical and laboratory features of infants with pneumonia demonstrated an elevated fatality rate in severely malnourished children compared to well-nourished infants [139]. A study examining the prevalence of respiratory infections to the prevalence of malnutrition in children under 5 years of age, found that acute upper respiratory infections were most prevalent among children with acute malnutrition. However, lower respiratory infections were most prevalent among children with either acute or chronic malnutrition. As most previous studies did not examine the effects of malnutrition on acute upper and lower respiratory infections separately, these results provide additional information to this complex area of study [123].

To identify potential differences in the etiology of pneumonia between children with and without severe malnutrition, Chisti *et al.* [130] conducted an excellent review study to quantify the degree by which moderate and severe degrees of malnutrition increase the mortality risk in pneumonia. They found that children with pneumonia and moderate or severe malnutrition showed a higher mortality risk. For severe malnutrition, reported relative risks ranged from 2.9 to 121.2; odds ratios ranged from 2.5 to 15.1. For moderate malnutrition, relative risks ranged from 1.2 to 36.5. These results show a significant association between moderate and severe malnutrition and mortality among children with pneumonia.

Furthermore, studies have demonstrated that pneumonia is more common among children with marasmic-kwashiorkor than among other types of malnourishment [140]. Additionally, in children under the age of 2 years, malnutrition is associated with a significant increase in ARI morbidity, also, severe pneumonia is associated to increase the mortality rate [141,142]. In a study performed with severely malnourished children, the mortality in children with Kwashiorkor was 13.4%. Mortality was 28% in children with marasmus and 48.3% in children with unclassified malnutrition. The main causes of death in children younger than 18 months of age were dehydration and pneumonia; in children from 19 to 60 months of age, it was pneumonia [143].

The data currently available suggest that the spectrum and frequency of causative agents of bacterial pneumonia in severely malnourished children may differ from that observed in children without severe malnutrition [130]. A study analyzing the etiology of pneumonia in severely malnourished children showed that the type and frequency of causative pathogenic microorganism differed from those reported in children without severe malnutrition [130].

*Streptococcus pneumoniae* and *Haemophilus influenzae* were the two microorganisms isolated most frequently from the blood, lung or pleural fluid from well-nourished (33%) and malnourished children (11%) with pneumonia [127,128]. However, according to Chisti *et al.* [130] *Klebsiella* ssp. and *S. aureus* were the most common causative organisms in severely malnourished children. These findings suggest that *Klebsiella* species and *S. aureus* are probably the main bacterial causes of pneumonia in malnourished children. Additionally, pathogenic viruses have been isolated from malnourished children with pneumonia. Although *Mycobacterium tuberculosis* was detected in 18% of malnourished children with pneumonia [140], the role of *Mycobacterium tuberculosis* presenting as an acute lower respiratory infection in severely malnourished children has not been well studied.

A prospective study of staphylococcal lower respiratory infections in children aged 1–48 months reported that 68% of the cases were diagnosed as bronchopneumonia. Of the 9.7% of patients in the study that that died, they were all malnourished children who did not receive antibiotics prior to disease presentation. Further, they all exhibited bronchopneumonia and *Staphylococcus aureus* positive blood cultures [144].

In the absence of an organized and effective immune response, antibiotics alone are usually incapable of eradicating bacterial pathogens [145]; therefore, antibiotics only have slight effect on early mortality from bacteremia and sepsis due to *Streptococcus pneumoniae* [146]. As we mentioned above, the innate immune response provides a first line of defense against infection. It has been estimated that the innate immune system provides protection against 98% of encountered pathogens [147]. The upper respiratory tract is the ecological niche for many bacterial species. *S. pneumoniae* is part of the commensal flora of the upper respiratory tract, as mentioned above. Together with *Haemophilus influenzae, Staphylococcus aureus, M. catarrhalis*, and various hemolytic streptococci, *S. pneumoniae* colonizes the nasopharyngeal tract [148].

Effective respiratory tract host defense against pathogens depends on the interaction of type-specific antibodies, complement, and neutrophils or other phagocytic cells [149,150]. If pathogens overcome these defenses and gain entry into the blood stream, systemic protection is mediated by anticapsular antibodies [151]. A reduced mucosal immune response might lead to persistent and recurrent colonization and subsequent infection, whereas an efficient local immune response to the pathogen eliminates colonization and prevents recolonization.

The pneumococcal cell wall is highly immunogenic, it is the cause of the intense inflammatory reaction that accompanies pneumococcal infection; it stimulates the influx of inflammatory cells, activates the complement cascade and induces cytokine production [152]. In general, the mucosal immune system develops faster than the systemic immune system, and functions from the age of 6 months. IgG and secretory IgA antibodies directed against capsular polysaccharides and surface-associated proteins have been observed in saliva of children under five years in response to colonization with *S. pneumoniae* [131].

There is evidence that the susceptibility of malnourished children to respiratory infections caused by encapsulated bacteria is due to defects in the production of IgG antibodies. However, malnutrition produces a profound depression on acquired cell-mediated immune competence, whereas humoral competence is less predictably affected. In contrast, in a recent study examined the effect of undernutrition on the humoral immune profile in children less than 60 months of age with pneumonia. The children were admitted to hospital with moderate-severe pneumonia, and undernutrition was associated with hypoalbuminemia and reduced humoral immune responses [153].

Immunoglobulin levels of malnourished children have been reported by various researchers to be comparable to well-nourished children; however, IgA levels are decreased in malnutrition [10]. In addition, previous report shows that the mean percentages of IL 4-producing T-cells are increased in malnourished children compared to well-nourished children [27]. Moreover, high levels of serum IL-4 have been found in malnourished children [154]. The high levels of IL-4 could contribute to the elevated levels of serum immunoglobulins reported in malnourished children [10]. The secretory IgA is a principal component of the mucosal immune response that protects the upper respiratory tracts against infection with pathogenic organisms; therefore, the diminished IgA levels observed in malnourished children may be responsible for diminished immune responses against respiratory infections.

In general, acute bacterial infections, such as *Streptococcus pneumonia*, are characterized by the predominance of neutrophils in the inflammatory reaction [155]. Chemokines are likely to play a major role in this type of immune response. A significant reduction of phagocytic capabilities and diminished killing capabilities of neutrophils in malnourished children has been reported [25]. Furthermore, in malnourished patients although there is a close-to-normal neutrophil chemotaxis and phagocytosis, minor defects in the generation of reactive oxygen intermediates and bacterial killing have been demonstrated [156]. Several investigators have demonstrated that malnutrition results in impaired macrophage phagocytosis, impaired superoxide anion production and reduced cytokine production [105]. Moreover, malnutrition has been shown to cause retarded macrophage differentiation [157].

However, protection against bacterial respiratory infections is also mediated by opsonin-dependent phagocytosis. Antibody-initiated complement-dependent opsonisation, which activates the classic complement pathway, is thought to be the main immune mechanism protecting the host against *S. pneumonia* infection [148]. In several studies, complement components were significantly lower in malnourished children [158,159]. In particular, C3 and factor B were depressed in malnourished patients [160]. Overall, complement production in response to infection and inflammation is inadequate in malnourished individuals [145]. These data suggest that a relative complement deficiency with decreased resistance to infections exists in malnourished children.

Malnourished mice infected with *Streptococcus pneumoniae* exhibited more lung injuries, impaired leukocyte recruitment and reduced antibody and cytokine production compared to well-nourished mice [100]. Diverse experimental evidence indicates that cytokines play an important role in the nutrition-infection complex [161]. Accordingly, an impairment of cytokine production has been reported in malnutrition [118,162].

Macrophages from protein-malnourished animals produced less TNF-α in response to infection [54,163]. Particularly, phagocytes in the respiratory tract of infected malnourished mice showed reduced TNF-α production and activity compared to infected well-nourished mice [101]. In contrast, other studies have shown that TNF-α production by PBMC from malnourished children did not differ compared to well-nourished children [164]. Consistent with this, IL-6 production in malnourished children was similar to well-nourished children. However, these results differ from those of Doherty *et al.* [165] who reported a diminished IL-6 production in severely malnourished children. In contrast, other studies found that IL-6 levels were significantly increased in the supernatants of phytohemagglutinin (PHA)-stimulated cultures from malnourished children compared to well-nourished children [166,167].

In a more recent study from our laboratory [27], production of IL-2, IFN-γ, IL-4 and IL-10) were evaluated in CD4+ and CD8+ T-cells. Peripheral blood CD4+ and CD8+ T-cells from malnourished children showed reduced IL-2 and IFN-γ production compared to well-nourished infected children. In contrast, an increase in Type 2 cytokine production was found. Decreased IL-2 and IFN-γ production has also been observed in other studies [168,169].

An important increase in the percentage of CD4+ and CD8+ IL 10-expressing cells is evident in malnourished children [27]. IL-10, which is produced by a variety of cells including T lymphocytes, B lymphocytes, and monocytes has been identified as a cytokine with important anti-inflammatory and immunosuppressive properties [170]. IL-10 is a major cause of ineffective anti-pathogen immune responses, as it inhibits many of the individual steps in antimicrobial immunity [171]. Therefore, IL-10 may be an important immunosuppressive factor related to the impaired immune response observed in malnourished children.

Altered levels of the proinflammatory cytokines granulocyte-macrophage colony stimulating factor (GM-CSF), IL-8 and IL-6 have been observed in the culture supernatants of PBMCs isolated from malnourished children. Specifically, GM-CSF levels were lower in malnourished children compared to well-nourished children, while IL-8 and IL-6 levels were higher in malnourished children compared to well-nourished children. These altered cytokine responses in PBMCs from malnourished children suggest severely impaired inflammatory responses [172].

When malnourished and well-nourished mice were challenged with *S. pneumoniae*, lung colonization and bacteremia were significantly greater in malnourished mice. The malnourished mice showed diminished numbers of leukocytes and neutrophils in the blood and in bronchoalveolar lavages. Although a moderate increase of leukocytes was observed after challenge with *S. pneumoniae*, there was a decrease of leukocytes on day 5 post-infection, most likely due affected cell release from the bone marrow [100]. Reduced capacity of leucocytes to kill ingested microorganisms and decreased ability of lymphocytes to replicate, coupled with lower concentrations of the cells responsible for cell-mediated immunity, results in higher morbidity due to infectious diseases [173]. Another probable explanation for the reduced bacterial clearance and increased mortality observed in malnourished children with pneumonia is defective alveolar macrophage function.

## 7. Malnutrition, Leptin and Bacterial Infections

Leptin has been identified to function as a prominent regulator of immune system activity, linking the function of the immunologic system to nutritional status [174–177]. Leptin is produced by adipose tissue in proportion to fat mass and is produced during the acute phase response. From an immunological point of view, leptin-deficient mice (ob/ob) display reduced cellularity in the spleen and thymus, and show increased susceptibility to infection.

Leptin levels normally increase acutely during infection and inflammation [178,179]. Moreover, it has been demonstrated that leptin plays an important role in T-cell mediated immune responses [180]. However, serum leptin levels are reduced in infected children who are severely malnourished [181,182]; therefore, diminished leptin concentrations in malnourished children may be involved in immune system dysfunction and increased susceptibility to infections [164]. Leptin has been shown to prevent lymphoid atrophy, reconstitute lymphoid cellularity [183] and to restore circulating lymphocyte populations during malnutrition [184]. Furthermore, macrophages obtained from leptin-deficient mice are deficient in phagocytosis, the addition of exogenous leptin to macrophages has been shown to augment macrophage phagocytosis, bacteriocidal activity and cytokine synthesis [174,176]. Infection has been shown to increase serum leptin levels *in vivo* [185].

Additionally, leptin administration restores a normal immune response. In 2007, Rodríguez *et al.* studied the effect of leptin on peripheral blood CD4+ and CD8+ T-cell cytokine production and activation in malnourished children. We demonstrated that leptin enhances IL-2 and IFN-γ secretion while inhibiting IL-4 and IL-10 production. These results demonstrate that human leptin can also modulate the activation of CD4+ and CD8+ T-cells from infected malnourished children [186].

The role of leptin in Gram-negative bacterial pneumonia was investigated by comparing the responses of normal mice and leptin-deficient mice following *Klebsiella pneumoniae* inoculation. As expected, normal mice displayed increased blood and lung leptin levels in response to bacterial pneumonia [187]. Compared to normal mice, leptin-deficient mice exhibited increased mortality and reduced bacterial clearance from the lung. This increased susceptibility to bacterial pneumonia in the leptin-deficient mice was associated with reduced alveolar macrophage phagocytosis of *K. pneumoniae in vitro*; importantly, *in vitro* alveolar macrophage phagocytosis function was restored by the addition of exogenous leptin [187]. Also, Leptin can augment IFN-γ synthesis during the course of bacterial pneumonia, which could enhance macrophage effector function. Therefore, leptin plays an important role in host defense against bacterial pneumonia.

These data indicate that leptin is an essential component of antibacterial host defense and those malnourished children are more susceptible to bacterial gastrointestinal and respiratory infections may be because they exhibit diminished levels of leptin.

## 8. Conclusions

Here, we focused on describing the interactions between malnutrition and immune system dysfunction and the determinants that provoke increased susceptibility to gastrointestinal and bacterial respiratory infections. PCM adversely affects the immune system; therefore malnutrition is considered the most common cause of immunodeficiency throughout the world. In synergy with infection, malnutrition contributes to 56% of all childhood deaths worldwide. The causes of malnutrition are multiple and complex and infections are a common precipitating factor. Acute gastrointestinal and respiratory infections are the most important causes of high morbidity and mortality among malnourished children and malnutrition is an important associated factor in these deaths. The studies described within this review provide evidence that the combination of several defective immune mechanisms synergistically inhibits the development of an adequate host immune response.

Particularly, defects in the innate immune response resulting from protein calorie malnutrition may contribute to the susceptibility of malnourished children to infection. Moreover, several studies have demonstrated that malnutrition severely impairs cytokine production, which may also be related to the impaired cell-mediated immunity in malnourished children.

The study of the interactions between malnutrition and the immune system may generate many practical and clinical applications. A better understanding of these interactions could contribute to more effective approaches to saving children’s lives. Additionally, strategies to more effectively reduce child malnutrition are urgently needed.
